# Incidence and prevalence of rheumatoid arthritis in Saskatchewan, Canada: 2001–2014

**DOI:** 10.1186/s41927-019-0077-4

**Published:** 2019-07-18

**Authors:** Bindu Nair, Regina Taylor-Gjevre, Liying Wu, Shan Jin, Jacqueline M. Quail

**Affiliations:** 1 College of Medicine, University of Saskatchewan, Saskatoon, Saskatchewan Canada; 2 Saskatchewan Health Quality Council, Saskatoon, Saskatchewan Canada

**Keywords:** Epidemiology, Rheumatoid arthritis, Administrative health database

## Abstract

**Background:**

Rheumatoid arthritis (RA) is a chronic inflammatory and destructive arthritis. Understanding the incidence and prevalence of RA within the province facilitates appropriate health care resource planning.

**Objective:**

To estimate the incidence/prevalence of RA over time for the overall provincial population, for specific age range categories, and for gender.

**Methods:**

Saskatchewan Provincial Administrative Health Databases (2001–2014) were utilized as data sources. Two RA case-definitions were employed: 1) > three physician billing diagnoses, at least one of which was submitted by a specialist (rheumatologist, general internist or orthopedic surgeon) within 2 years; 2) > one hospitalization diagnosis (ICD-9-CM code-714, and ICD-10-CA code-M05). Data from these definitions were combined to identify incident and prevalent RA cases. Using this data, annual incidence and prevalence rates were calculated for the provincial population, specified age range categories and gender categories.

**Results:**

The number of RA cases meeting the case definition increased from 3731 to 6223 over the study period. The incidence of RA disease demonstrated variation within the study period with age and sex adjusted incidence ranging from 33.6 (95% CI 29.9–37.6) per 100,000 to 73.1 (95% CI 67.6–79.0) per 100,000. The prevalence of RA increased over time from 482 (95% CI 466.7–497.7) per 100,000 in 2001–2002 to 683.4 (95% CI 666.6–700.6) per 100,000 in 2014–2015. Both incidence and prevalence rates rose with increasing age. Women were found to have higher incidence and prevalence rates compared to men.

**Conclusion:**

In Saskatchewan, the overall prevalence of RA is rising while there has been variability in the incidence.

## Background

Timely identification of rheumatoid arthritis (RA) is crucial in order to initiate therapy and limit joint damage. Control of inflammatory disease and clinical remission are now achievable standards particularly with the advances in immunosuppressive biologic therapies. [1]. However, despite guidelines for the management of RA, it is recognized that there is an underutilization of appropriate treatments [2, 3]. Identification of groups that are at higher risk for greater disease burden or have decreased access to health care services would be invaluable to inform the delivery of health care resources. In Saskatchewan, Canada, RA patients residing outside major urban areas have reported concerns with decreased access to specialists and allied health professionals [4]. Recognition of the time trends of incidence and prevalence of the disease would be important to further advise future healthcare needs for this population.

The annual incidence of RA has been reported to be approximately 0.5 per 1000 people per year in the United States [5]. A prevalence of RA of 1–2% is reported and the epidemiology differs depending on ethnic and geographic distribution [6]. A greater prevalence has been reported in certain Indigenous populations [7]. Epidemiologic variations in incidence and prevalence of RA have been observed with a general decreasing trend in incidence over the years reported by several investigators [8–11]. Conversely, prevalence estimates of rheumatoid arthritis have been observed to be increasing by other researchers, with a suggestion that this phenomenon may be secondary to increased survival of RA patients [12]. In 2010, the American College of Rheumatology (ACR)/European League Against Rheumatism (EULAR) published classification criteria for RA with the aim of allowing earlier identification of the disease than the 1987 classification criteria [13]. Comparison studies on the sensitivity and specificity of case identification of the 2010 and the 1987 RA classification criteria have found them to be approximately similar; one study showing that the 2010 criteria appeared to identify at baseline similar rates of RA as the 1987 criteria identified cumulatively over 5 years [14].

Saskatchewan is a province in Canada with a population of 1,132,300 people in 2015 over a land area of 588,239.21 km (http://www.statcan.gc.ca/tables-tableaux/sum-som/l01/cst01/demo02a-eng.htm). Two-thirds of the population resides in urban areas and the majority of people live in the southern third of the province. Saskatoon and Regina are the two major cities where the tertiary medical care centers for the province are located as well as where all the province’s subspecialist rheumatologists practice. The objectives of this study were 1. To map the incidence and prevalence of RA within Saskatchewan over time and 2. To determine if the estimates vary across age or gender. Recognition of diverging incidences and prevalence of RA may serve as the basis to develop a policy framework for health care delivery.

## Methods

### Setting and design

Saskatchewan is one of three Prairie Provinces in Western Canada. The majority of Saskatchewan residents receive provincial health care benefits. The rest of the population (< 1%) would include federally insured persons such as federal prison inmates, Royal Canadian Mounted Police (RCMP) and military personnel. These federally insured groups would have information captured in data collected around hospital use but not in other data sources employed in this study. Additionally, First Nations and Metis peoples whom have treaty relationships with the federal government, termed ‘Registered Indians’ (RI) and who comprise 15.6% (http://www12.statcan.gc.ca/nhs-enm/2011/as-sa/fogs-spg/Pages/FOG.cfm?lang=E&level=2&GeoCode=47) of the provincial population, receive some of their health benefits from the Federal Government. Relevant data from these populations with Federal treaty relationships was incorporated within the data sources employed in this study.

A Saskatchewan provincial population-based cohort study was undertaken to evaluate incidence and prevalence rates for RA between Fiscal Year 2001–02 (FY0102) and FY1415.

### Subjects and data sources

This retrospective, population-based cohort study was achieved by employing the Saskatchewan Provincial Health Administrative Databases. All data available, spanning from April 1, 1996 to March 31, 2015, were used to identify the cohort. Provincial Health Administrative Databases utilized for this study included: the *Discharge Abstract Database*, the physician *Medical Services Database*, the *Person Health Registration System*, and the *Vital Statistics Registry*. These various data sources can be linked anonymously through unique personal health insurance numbers.

The *Discharge Abstract Database* incorporates detailed hospitalization related data. Until March 31, 2001, prior to the study time period, diagnoses were recorded in compliance with the International Classification of Diseases 9th revision (ICD-9). Subsequently, the International Classification of Diseases, 10th revision, Canadian Version (ICD-10-CA) was introduced. During the 12-month period from April 1, 2001 until March 31, 2002, approximately 90% of ICD codes recorded are ICD-10-CA with the remaining 10% being ICD-9. Subsequent to April 1, 2003 virtually all codes were recorded in the ICD-10-CA format. Between 3 and 16 diagnoses are captured in each record prior to the introduction of ICD-10-CA, and up to 25 diagnoses are captured subsequently. The database provides: detailed diagnostic information including the primary admission diagnosis, as well as co-morbidity diagnoses and diagnoses related to complications arising during the hospitalization.

The *Medical Services Database* provides data on physician services. Physicians who are paid on a fee-for-service basis submit billing claims to the provincial health ministry. A single diagnosis using three digit ICD-9 codes is recorded on each claim. Physicians who are salaried are generally required to submit billing claims for administrative purposes, a practice known as shadow billing.

The *Person Health Registration System* captures characteristics of each insured individual including their age, sex, location of residence, and dates of coverage within the provincial health insurance plan.

The *Vital Statistics Registry* holds information on all births and deaths within the province.

### Cohort case definition

#### Rheumatoid arthritis

A previously validated algorithm for administrative data was employed in the identification of people with RA for this cohort [15]. Individuals were identified as having RA if they had three or more physician services claims for RA (ICD-9 code: 714), at least one of which was made by a rheumatologist, general internal medicine specialist or orthopedic surgeon, within a 2 year period, or if they had one or more hospitalizations with a diagnosis of RA (ICD-9 code: 714, ICD-10 codes: M05, M06) in any of the up to 25 diagnosis fields. When an individual met both the physician visit and hospitalization criteria, the earliest occurrence was taken as the index date of diagnosis. For inclusion in this cohort, individuals were required to be age 18 or older on the index date of their RA diagnosis and have uninterrupted health insurance coverage (i.e. a gap of no more than three consecutive days in coverage) from the date of their diagnosis until March 31, 2015 or their exit from the cohort.

To distinguish incident from prevalent RA, a lead-time of 2 years was used based on the clinical judgement that a person is unlikely to go more than 2 years without seeking medical attention for their newly developing RA. Individuals having 2 or more years of insurance coverage prior to their diagnosis of RA were identified as having incident RA. Anyone with less than 2 years of health insurance prior to their diagnosis with RA was included in the cohort to ensure prevalence estimates in subsequent years were accurate. They were included in incidence estimates, although they were flagged to determine the potential overestimation of the incidence of RA.

#### Death

The date of death was identified from the Vital Statistics Registry. An individual who died between April 1 and March 31 of the following year was described as having died in that fiscal year.

### Co-variates

Descriptive variables were identified for each member of the cohort. All variables were determined on the day of RA diagnosis. These included: age (categories: 18- < 45, 45- < 55, 55- < 65, 65- < 75, 75 and more years), sex (male, female), insurance coverage (< 2 years, 2 and more years). Age, sex, and insurance coverage were obtained from the Person Health Registration System.

### Statistical analysis

All analyses were performed at the Saskatchewan Health Quality Council using SAS© statistical software, version 9.3 (SAS Institute Inc. SAS. Cary, NC: SAS Institute Inc.; 2007). The FY0102 population data was used as the reference for directing standardization of subsequent years. Cell size count data of less than six was suppressed as per Saskatchewan Health Quality Council policy for protection of patient confidentiality.

The incidence rate of RA per 100,000 population at risk (PAR) was calculated for each fiscal year. As an example, in FY0102 the numerator is the number of people alive on April 1, 2001 who were diagnosed with RA between April 1, 2001 and March 31, 2002, inclusive. The denominator (i.e., the population at risk to develop RA) includes all individuals aged 18 years or older on April 1, 2001 with at least 1 day of health insurance coverage within the fiscal year after removing individuals with prevalent RA.

The prevalence rate of RA per 100,000 PAR was calculated for each fiscal year (FY). As an example, in FY0102, the numerator is the number of people alive on April 1, 2001 who were diagnosed with RA *prior* to April 1, 2001. The denominator includes all individuals aged 18 years or older on April 1, 2001 with at least 1 day of health insurance coverage within the fiscal year. Prevalent cases were carried forward each year unless they died or had a gap in their health coverage longer than 3 days.

Crude and adjusted annual incidence and prevalence rates and 95% confidence intervals (95% CI) were calculated for FY0102 to FY1415. Rates were adjusted to the age and sex distribution of the Saskatchewan population standard from fiscal year 2001/02. Annual rates were also stratified by sex and also by age category. Sex-stratified rates were standardized for age. Age category rates were standardized for sex.

This study was approved by the University of Saskatchewan Biomedical Research Ethics Board.

## Results

We identified 9488 people with RA between April 1, 2001 and March 31, 2015 (Table 1). A total of 3731 were diagnosed prior to FY0102 and so were identified as having prevalent RA in that year, and 5757 were diagnosed with RA between April 1, 2001 and March 31, 2015. The proportion of incident RA cases with less than 2 years of health insurance prior to their diagnosis in each fiscal year ranged from 3.3 to 8.2%, with a mean of 5.9%.

**Table 1:**
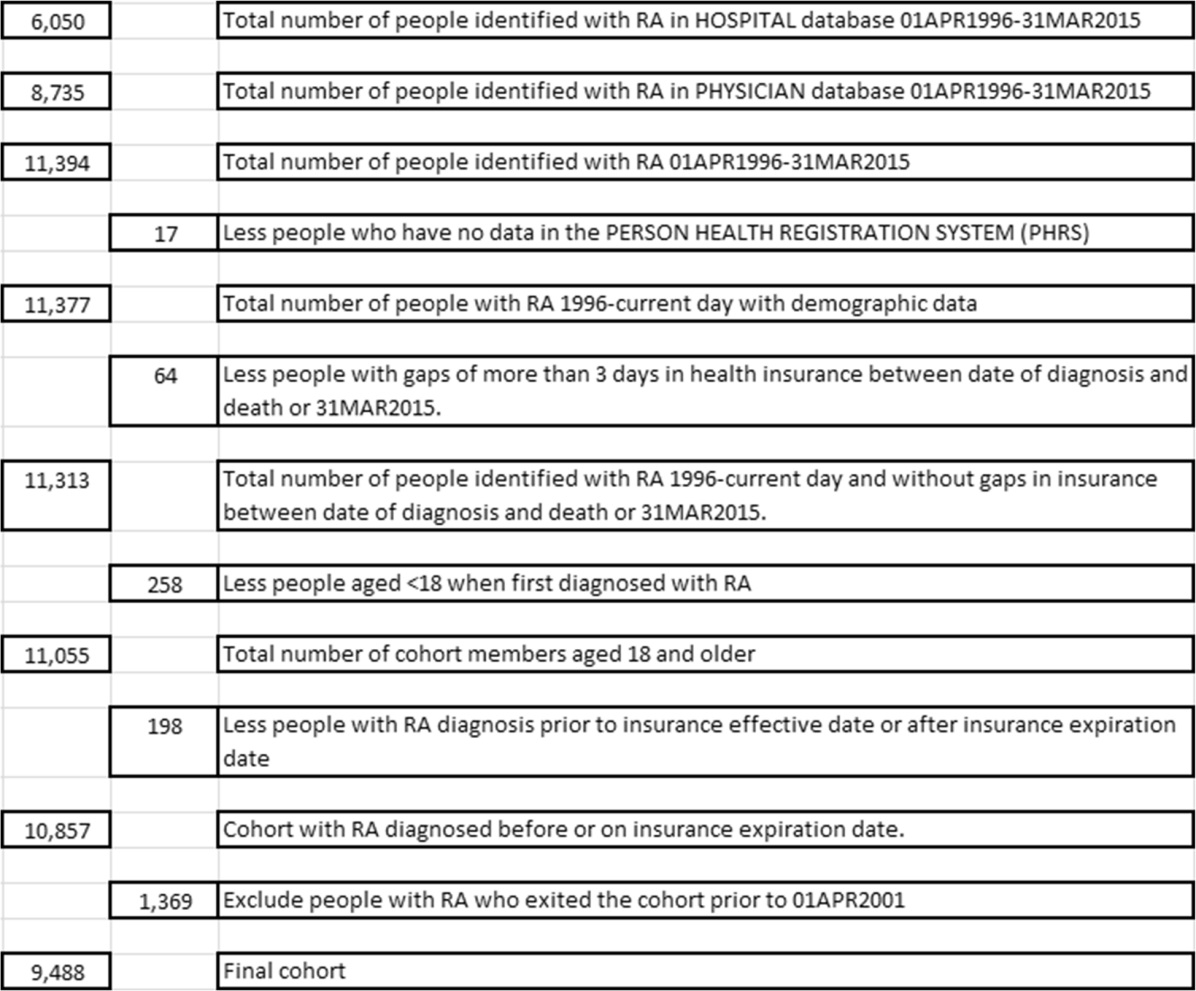
Description of cohort creation

**Fig. 1:**
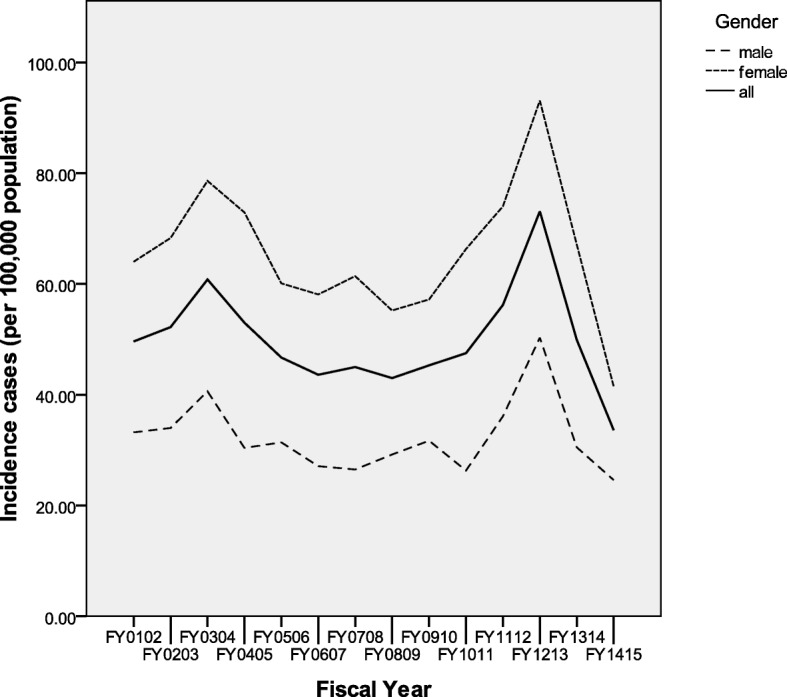
Provincial RA Incidence rates (age adjusted) per fiscal year

Incident RA cases varied from a low of 304 cases in FY1415 to a high of 637 cases in FY1213. Corresponding incidence rates adjusted for age and sex varied from 33.7 cases/100,000 to 73.1 cases/100,000 PAR (Table 2). There did not appear to be any consistent trend toward increase or decrease in age adjusted incidence rates for either men or women over this time period (Fig. 1). Women had higher incidence rates than men. Variations in incidence were observed with a single year peak in FY0203 and another ascending trend from FY1112 peaking in FY1213 with subsequent reversal the following year.

**Table 2:** Crude and adjusted incidence/prevalence rates for rheumatoid arthritis in Saskatchewan

Provincial rates per fiscal year (FY)	Incident cases	Population denominator	Crude incidence rate	Adjusted^a^ Incidence Rate (95% CI)	Prevalent cases	Population denominator	Crude prevalence rate	Adjusted^a^ Prevalence Rate (95% CI)
FY0102	382	772,025	49.5	49.6 (44.7,54.8)	3731	775,756	481.0	482.0 (466.7497.7)
FY0203	406	775,969	52.3	52.2 (47.3,57.6)	3905	779,874	500.7	499.6 (484.1515.5)
FY0304	473	771,409	61.3	60.8 (55.5,66.6)	4098	775,507	528.4	523.0 (507.2, 539.2)
FY0405	416	778,387	53.4	53.0 (48.0,58.3)	4378	782,765	559.3	553.3 (537.0,569.8)
FY0506	370	784,134	47.2	46.7 (42.0, 51.7)	4588	788,722	581.7	573.4 (557.0,590.2)
FY0607	346	780,217	44.3	43.6 (39.1, 48.4)	4718	784,935	601.1	587.4 (570.8604.4)
FY0708	364	796,132	45.7	45.0 (40.5, 49.9)	4828	800,960	602.8	591.2 (574.7608.0)
FY0809	354	814,207	43.5	43.0 (38.7, 47.7)	4954	819,161	604.8	596.8 (580.4613.6)
FY0910	376	820,168	45.8	45.3 (40.8, 50.1)	5091	825,259	616.9	607.6 (591.1624.5)
FY1011	402	838,366	48.0	47.5 (43.0, 52.4)	5243	843,609	621.5	614.4 (597.9631.2)
FY1112	484	856,532	56.5	56.2 (51.3, 61.4)	5383	861,915	624.5	620.4 (604.0,637.1)
FY1213	637	867,023	73.5	73.1 (67.6, 79)	5624	872,647	644.5	640.6 (624.0,657.5)
FY1314	443	887,734	49.9	49.9 (45.4, 54.8)	6021	893,755	673.7	673.5 (656.7690.7)
FY1415	304	905,624	33.6	33.6 (29.9, 37.6)	6223	911,847	682.5	683.4(666.6–700.6)

Rates per 100,000 population; *CI* Confidence intervals

^a^ Adjusted for sex and age as a continuous variable

**Fig. 2:**
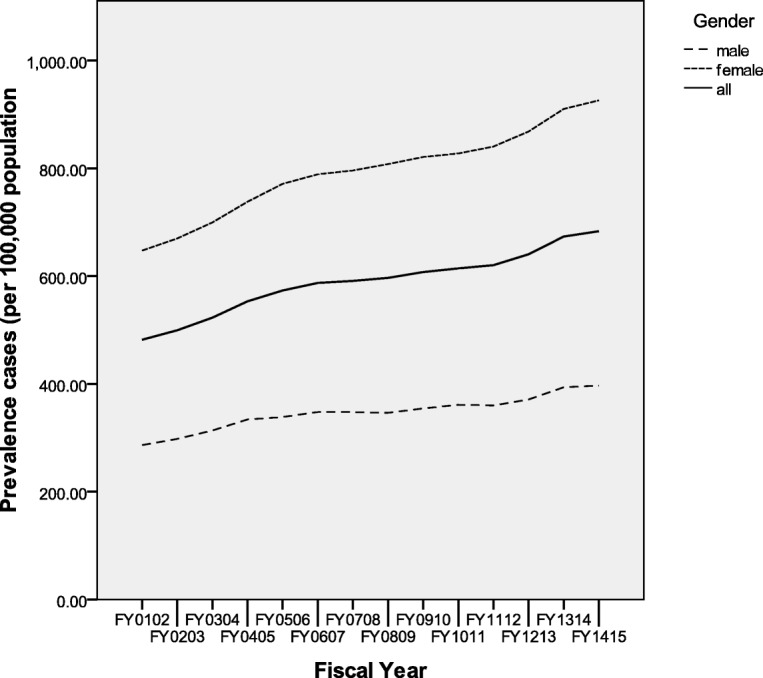
Provincial RA Prevalence rates (age adjusted) per fiscal year

The population with prevalent RA increased from 3731 in FY0102 to 6223 in FY1415, with corresponding increase in age and sex adjusted prevalence rates from 482.0 cases/100,000 PAR (0.5%) to 683.4 cases/100,000 PAR (0.7%) (Table 2). A steadily increasing trend in prevalence is observed over the time period for the population overall and separately for both genders (Fig. 2). There was higher RA prevalence in women compared to men.

**Fig. 3:**
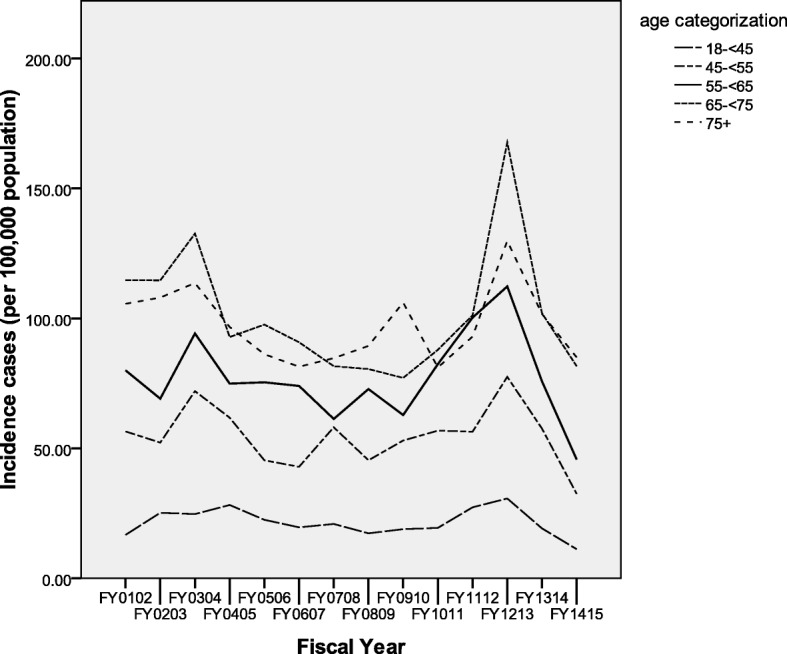
Age category incidence rates (sex adjusted) per fiscal year

**Fig. 4:**
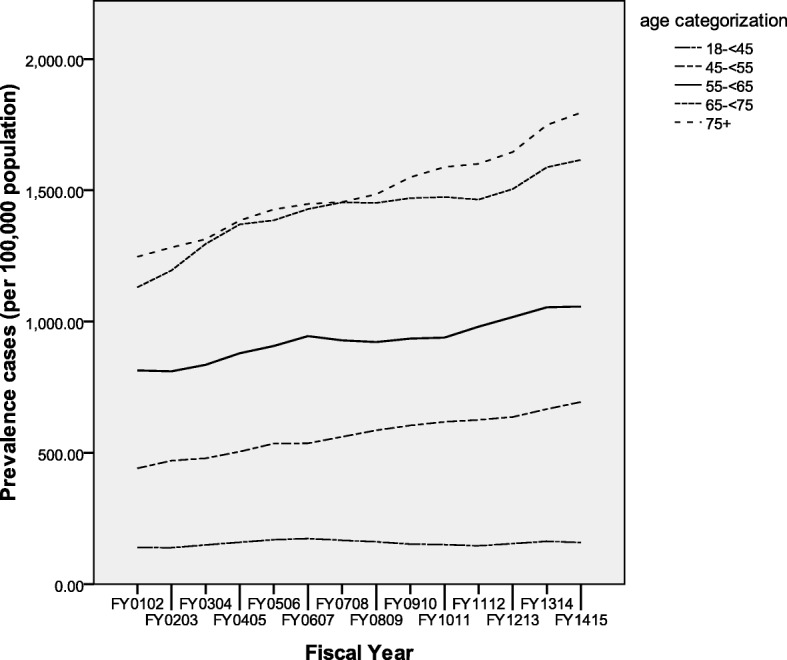
Age category prevalence rates (sex adjusted) per fiscal year

Figure 3 illustrates the RA incidence rates (adjusted for sex) by age categorization over the study time period. The five age categories are represented. The lowest incidence rate was observed in the 18- < 45 years group, with steadily increasing rates for each of the ascending age brackets. There was overlap between the 65- < 75 and 75 and more year groups, with the 65- < 75 year group generally having higher annual rates. Prevalence rates for individual age categories revealed similar findings over time, with distinct layers of prevalence ascending with age (Fig. 4). Again some overlap between the two most advanced age groups was observed, with overall the 75 and older group generally exhibiting the highest prevalence within this study period.

## Discussion

This study of the trends in incidence and prevalence of RA in the province of Saskatchewan from 2001 to 2015 has shown variations in the incidence and an overall rising prevalence of the disease. The prevalence of RA increasing over time is similar to results from other recent epidemiologic studies in Canada and the United States [12, 16]. The change in prevalence may be secondary to changes in treatment strategy for rheumatoid arthritis patients over the years with an increased use of immunosuppressive medications [17]. The Treat-to-Target approach has resulted in increased awareness of the importance of early diagnosis and treatment of rheumatoid arthritis to improve clinical outcomes [1] which may contribute to heighted recognition of the disease and need for longitudinal management. A recent study in contrast has reported that while the prevalence of RA in the United Kingdom increased from 1990 to 2005, it was noted that from 2005 to 2014 the prevalence of RA had decreased [18].

Our study has also demonstrated peaks of increased incidence from 2003 to 2004 and from 2011 to 2014. While other studies have similar results with an increase in the incidence of RA [16, 19], there are also differing reports regarding the incidence of the disease not showing any change or having a decrease incidence [11, 12, 18]. There are probably multiple factors that may be associated with our results, some which may be generalizable to other regions and some that are local. An escalated incidence in RA seems to have followed the publication of the revised American College of Rheumatology/European League Against Rheumatism Classification Criteria for RA [13]. The newer classification criteria aimed to identify RA patients at an earlier stage in the disease as compared to earlier classification criteria. Dissemination and implementation of the newer classification criteria by health professionals may have resulted in an increased awareness of early diagnosis of RA with more incident cases being identified. The periods of heightened incidence may also relate to times when new rheumatologists started their practice in the province and with resulting improvement of patient access to rheumatology subspecialty care. However the Saskatchewan Provincial Health Databases do not distinguish submission of claims by rheumatologists from other internal medicine specialists and therefore we would be unable to determine if the incidence changes relate to difference of case entry over time by different specialties or subspecialties.

The role of environmental factors may be considered in examining the changing epidemiology of RA in the province. Cigarette smoking has been associated with the risk of developing RA in both genders [20]. While smoking trends have decreased, the rate of decline has been less in women and thereby possibly contributing to the change in incidence [21]. Cigarette smoking has demonstrated to be of higher prevalence in certain Canadian First Nations populations that research has also shown similar trends with the prevalence of rheumatic diseases such as RA [22–24].

Administrative health databases are valuable for epidemiological studies as they contain a large amount of data that has been systematically collected for a population over a period of time. However these health databases were not designed for the intent of research. We do acknowledge that a limitation of this study is the reliance on a case definition of RA using administrative health database codes rather than using the clinical and serological classification criteria which may cause either overestimation or underestimation of the disease. Thus the estimates of incidence and prevalence that we found for the province of Saskatchewan may not be able to be generalized to other populations.

## Conclusions

In conclusion, we observed that in Saskatchewan, the prevalence of RA is rising and there has been variability in the incidence of RA. Women had overall higher incidence and prevalence rates for RA compared to men. Factors associated with these results may be due to the growing awareness of the need to identify and treat RA cases early in the disease. Future research directions include looking at the mortality trends and healthcare service utilization of RA in this population.

## Data Availability

Data for this study was accessed by the Saskatchewan Health Quality Council under data sharing agreements with the Saskatchewan Ministry of Health and eHealth Saskatchewan. These agreements do not permit the sharing of record-level data outside of the secure data area at the Saskatchewan Health Quality Council.

## Abbreviations

ACR: American College of Rheumatology
CI: Confidence intervals
EULAR: European League Against Rheumatism
FY: Fiscal Year
ICD-10-CA: International Classification of Diseases 10th Revision, Canadian Version
ICD-9: International Classification of Diseases 9th Revision
km: Kilometers
PAR: Population at risk
RA: Rheumatoid arthritis
RCMP: Royal Canadian Mounted Police
RI: Registered Indians
SAS: Statistical Analysis System

## References

[1] Stoffer M, Schoels M, Smolen J, Aletaha D, Breedveld F, Burmester G, Bykerk V, Dougados M, Emery P, Haraoui B, Gomez-Reino J, Kvien T, Nash P, Navarro-Compan V, Scholte-Voshaar M, van Vollenhoven R, van der Heijde D, Stamm T (2016). Evidence for treating rheumatoid arthritis to target: results of a systematic literature search update. Ann Rheum Dis.

[2] Bykerk V, Akhavan P, Hazlewood G, Schieir O, Dooley A, Haraoui B, Khraishi M, Leclercq S, Legare J, Mosher D, Pencharz J, Pope J, Thomson J, Thorne C, Zumer M, Bombardier C (2012). Canadian Rheumatology Association recommendations for pharmacological management of rheumatoid arthritis with traditional and biologic disease modifying antirheumatic drugs. J Rheumatol.

[3] Lacaille D, Anis A, Guh D, Esdaile JM (2005). Gaps in care for rheumatoid arthritis: a population study. Arthritis Rheum.

[4] Nair BV, Schuler R, Stewart S, Taylor-Gjevre RM (2016). Self-reported barriers to healthcare access for rheumatoid arthritis patients in rural and northern Saskatchewan: a mixed methods study. Musculoskeletal Care.

[5] Alamanos Y, Voulgari PV, Drosos AA (2006). Incidence and prevalence of rheumatoid arthritis based on the 1987 American College of Rheumatology criteria: a systematic review. Semin Arthritis Rheum.

[6] Spector (1990). Rheumatoid arthritis. Rheum Dis Clin N Am.

[7] Peschken C, Esdaile J (1998). Rheumatic diseases in North America’s indigenous people. Semin Arthritis Rheum.

[8] Hochberg MC (1990). Changes in the incidence and prevalence of rheumatoid arthritis in England and Wales, 1970-1982. Semin Arthritis Rheum.

[9] Maradit K, Crowson C, Gabriel S (2004). Rochester epidemiology project: a unique resource for research in the rheumatic diseases. Rheum Dis Clin N Am.

[10] Doran M, Pond G, Crowson C (2002). Trends in incidence and mortality in rheumatoid arthritis in Rochester, Minnesota over a forty-year period. Arthritis Rheum.

[11] Kaipiainen-Seppanen O, Kautiainen H (2006). Declining trend in the incidence of rheumatoid factor-positive rheumatoid arthritis in Finland 1980-2000. J Rheumatol.

[12] Widdifield J, Paterson J, Bernatsky S, Tu K, Tomlinson G, Kuriya B, Thorne J, Bombardier C (2014). The epidemiology of rheumatoid arthritis in Ontario, Canada. Arthritis Rheumatol.

[13] Aletaha D, Neogi T, Silman A, Funovits J, Felson D, Bingham C, Birnbaum N, Burmester G, Bykerk V, Cohen M, Combe B, Costenbader K, Dougados M, Emery P, Ferraccioli G, Hazes J, Hobbs K, Huizinga T, Kavanaugh A, Kay KT, Laing T, Mease P, Menard H, Moreland L, Naden R, Pincus T, Smolen J, Stanislawska-Bjernat E, Symmons D, Tak P, Upchurch K, Vencovsky J, Wolfe F, Hawker G (2010). 2010 rheumatoid arthritis classification criteria: an American College of Rheumatology/European league against rheumatism collaborative initiative. Ann Rheum Dis.

[14] Humphreys J, Verstappen S, Hyrich K, Chipping J, Marshall T, Symmons D (2013). The incidence of rheumatoid arthritis in the UK: comparisons using the 2010 ACR/EULAR classification criteria and the 1987 ACR classification criteria. Results from the Norfolk Arthritis Register. Ann Rheum Dis.

[15] Widdifield J, Bernatsky S, Paterson J, Tu K, Ng R, Thorne J, Pope J, Bombardier C (2013). Accuracy of Canadian health administrative databases in identifying patients with rheumatoid arthritis: a validation study using the medical records of rheumatologists. Arthritis Care Res.

[16] Myasoedova E, Crowson C, Kremers H, Therneau T, Gabriel S (2010). Is the incidence of rheumatoid arthritis rising? Results from Olmsted County, Minnesota, 1955-2007. Arthritis Rheum.

[17] Kim S, Yelin E, Tonner C, Solomon D (2013). Changes in use of disease-modifying antirheumatic drugs for rheumatoid arthritis in the United States during 1983-2009. Arthritis Care Res.

[18] Abhishek A, Doherty M, Kuo C, Mallen C, Zhang W, Grainge M (2017). Rheumatoid arthritis is getting less frequent – results of a nation-wide population-based cohort study. Rheumatology (Oxford).

[19] Pedersen J, Svendsen A, Horslev-Petersen K (2007). Incidence of rheumatoid arthritis in the southern part of Denmark from 1995 to 2001. Open Rheumatol J.

[20] Silman A, Newman J, MacGregor A (1996). Cigarette smoking increases the risk of rheumatoid arthritis. Results from a nationwide study of disease-discordant twins. Arthritis Rheum.

[21] Tomar S (2003). Trends and patterns of tobacco use in the United States. Am J Med Sci.

[22] Riediger N, Lukianchuk V, Lix L, Elliott L, Bruce S (2015). Between a rock and a hard place: smoking trends in a Manitoba first nation. Can J Public Health.

[23] Barnabe C, Jones C, Bernatsky S, Peschken C, Voaklander D, Homik J, Crowshoe L, Esdaile J, El-Gabalawy H, Hemmelgarn B (2017). Inflammatory arthritis prevalence and health services use in the first nations and non-first nations populations of Alberta, Canada. Arthritis Care Res.

[24] Broten L, Aviña-Zubieta J, Lacaille D, Joseph L, Hanly JG, Lix L, O'Donnell S, Barnabe C, Fortin P, Hudson M, Jean S, Peschken C, Edworthy SM, Svenson L, Pineau C, Clarke A, Smith M, Bélisle P, Badley M, Bergeron L, Bernatsky S (2014). Systemic autoimmune rheumatic disease prevalence in Canada: updated analyses across 7 provinces. J Rheumatol.

